# Shared decision-making and detection of comorbidities in an online acromegaly consultation with and without the Acromegaly Disease Activity Tool ACRODAT^®^ using the simulated person approach

**DOI:** 10.1007/s11102-024-01460-6

**Published:** 2024-09-25

**Authors:** Anna Lena Friedel, Lisa Schock, Sonja Siegel, Angelika Hiroko Fritz, Nicole Unger, Birgit Harbeck, Philipp Dammann, Ilonka Kreitschmann-Andermahr

**Affiliations:** 1 Department of Neurosurgery and Spine Surgery, Member of ENDO-ERN, University Hospital Essen, Essen, Germany; 2 Institute for Medical Education, University of Duisburg-Essen, Essen, Germany; 3 Center for Translational Neuro- & Behavioral Sciences (C-TNBS), University of Duisburg-Essen, Essen, Germany; 4 German Cancer Consortium (DKTK) Partner Site, University Hospital Essen, Essen, Germany; 5 Simulation Persons Program, Medical Faculty, University of Duisburg-Essen, Essen, Germany; 6 Department of Endocrinology, Diabetes and Metabolism, Member of ENDO-ERN, University Hospital Essen, Essen, Germany; 7 III. Department of Medicine, University Medical Center Hamburg-Eppendorf, Hamburg, Germany; 8 MVZ Amedes Experts, Endocrinology, Hamburg, Germany; 9 Cognitive Neuropsychology, Department of Neurosurgery and Spine Surgery, University Hospital Essen, Hufelandstraße 55, D-45147 Essen, Germany

**Keywords:** Acromegaly, ACRODAT^®^, Shared decision-making, Simulation person, Comorbidity

## Abstract

**Objective:**

A patient-centered approach to the management of acromegaly includes disease activity control, shared decision-making and identification of comorbidities. The Acromegaly Disease Activity Tool (ACRODAT^®^) is intended to assist physicians in providing such holistic management. The present study investigated this claim using the simulated person (SP) approach.

**Methods:**

We studied patient-doctor interaction via online video consultation in a randomized prospective study design with SPs trained to simulate a specific acromegaly profile. We analyzed the proportion of conversation time devoted to health content and the specific acromegaly and comorbidity relevant categories mentioned in the conversation. We collected physicians’ feedback on the usefulness of ACRODAT^®^, SPs subjective perception of the quality of the conversation and compared consultations with and without ACRODAT^®^ using a qualitative approach.

**Results:**

The sample (*N* = 30) consisted of endocrinologists treating patients with acromegaly in Germany. For SP-physician interactions (*N* = 60), the proportion of time spent on conversation content (e.g. IGF-I, quality of life) was distributed according to the focus of the patient profile. Comorbidities were less well identified than the need for a change in therapy. Only 18.3% of the SPs were actively asked to participate in the decision-making process. ACRODAT^®^ did not lead to any significant differences in the course of the discussion.

**Conclusions:**

Shared decision-making was underrepresented in this SP-physician interaction in acromegaly management. Physicians adapted the content of the discussion to the SP’s needs, but did not adequately address comorbidities. According to the analysis criteria used, ACRODAT^®^ did not contribute to a more holistic patient management in the present study.

**Supplementary Information:**

The online version contains supplementary material available at 10.1007/s11102-024-01460-6.

## Introduction

In recent years, there has been a strong emphasis on patient-centeredness and active participation in the healthcare process. This paradigm-shift away from a more paternalistic patient-doctor relationship also concerns patients with acromegaly. The rare chronic neuroendocrine disorder is usually caused by a growth hormone-secreting pituitary adenoma resulting in an overproduction of growth hormone (GH) and elevated levels of insulin-like growth factor-I (IGF-I) [1, 2]. Clinical symptoms of the disease include disproportionate skeletal growth, coarse facial features, overgrowth of soft tissue, metabolic, respiratory and cardiovascular comorbidities, bone and joint diseases or sleep apnea [1]. Acromegaly patients have a strong desire to play an active role in their disease management and want a holistic approach to their medical care [3]. Since only few physicians have extensive experience in managing this rare disease and given the limited time available for medical consultations [4], it can be easily assumed that the illness and its comorbidities as well as symptom burden and psychosocial well-being of acromegaly patients cannot be adequately addressed during a typical specialist visit.

To counteract these existing shortcomings, the Acromegaly Disease Activity Tool (ACRODAT^®^) has been developed by a panel of internationally renowned endocrinologists and sponsored and owned by Pfizer Inc [5–7]. ACRODAT^®^ focuses on five disease-specific parameters (IGF-I, tumor size, comorbidities, acromegaly related symptoms, and quality of life, QoL) to estimate disease activity in patients with acromegaly. It provides the treating physician with a patient profile grading each of the five parameters (Grade 1: adequately controlled disease; Grade 2: mild disease activity; Grade 3: significant disease activity). ACRODAT^®^ is intended to assist physicians in making treatment decisions in terms of biochemical control of acromegaly, but also to enable a more holistic approach to the management of these patients with graphical representations giving an overview of QoL and symptom burden over the course of treatment.

To support the clinical assessment and management of acromegaly, there are *patient-reported outcome and / or experience measures*, such as the Acromegaly Quality of Life Questionnaire (AcroQoL) or the Pain Assessment Acromegaly Symptom Questionnaire (PASQ), the Acromegaly Treatment Satisfaction Questionnaire (ACRO-TSQ) and the Treatment Adherence, Satisfaction and Knowledge Questionnaire (TASK-Q). These instruments focus on the subjective perception of the disease with its physical and psychological signs and symptoms, the resulting disease burden, personal knowledge and satisfaction concerning the own treatment, the impact of that treatment and the patient’s adherence [8–10]. The *clinician-reported outcome measures* are ACROSCORE, SAGIT^®^ and ACRODAT^®^. The former aims to identify patients with acromegaly at the earliest possible stage, thus only signs, symptoms and comorbidities with early onset are included. SAGIT^®^ and ACRODAT^®^ were developed to measure disease activity, with the advantage of a complete evaluation of pathology, as it includes clinical aspects, biochemical values, and tumor characteristics. ACRODAT^®^ stands out here, as mentioned above, it also addresses one aspect of the *patient-reported experience measures*, namely QoL [7, 8].

Guidelines stated, that assessment of QoL (as an aspect of *patient-reported outcome and / or experience measures*) can be helpful in identifying specific factors for follow-up, although concordance with biochemical measures is limited. In addition, they recommend the use of *clinician-reported outcome instruments* as they can be helpful in assessing disease activity [11, 12]. SAGIT^®^ and ACRODAT^®^ are explicitly mentioned as useful instruments several times [13, 14].

ACRODAT^®^’s claim, that patients treated with the help of this software tool will achieve better biochemical disease control can easily be examined in a randomized and standardized prospective study design. However, it is much more difficult to verify whether the tool is helpful in structuring patient-doctor interaction in the sense of shared decision-making [15], bringing to light patient-centered factors that would be left unspoken in a “normal” doctor’s visit and/or leading to different or additional treatment decisions. A promising approach to answering the latter question is the simulated person (SP) approach, increasingly used in medical student education and clinical research [16–18]. This involves a trained actor (termed a SP), who has rehearsed a specific medical history based on a standardized script, interacting with medical staff in a specific healthcare setting. One important outcome of interest in such an interaction is the communication skills of health professionals. Accordingly, we decided to use a cross-sectional, multi-center, control-group design to investigate the influence of ACRODAT^®^ on decision-making and patient-doctor interaction via online video consultation with SPs who have been trained to simulate a patient with a specific acromegaly profile. A further goal was to collect feedback on the usefulness of ACRODAT^®^ from the physicians as well as the subjective individual perception of the SPs regarding the quality of the conversation in relation to the use of ACRODAT^®^.

## Methods

### Sample

Physicians in specialist training to become endocrinologists and board-certified endocrinologists treating patients with acromegaly in Germany were the target population for the proposed study. Physicians already working with ACRODAT^®^ on a regular basis or failing to provide written informed consent were not included in the study.

### Case scenarios

**Fig. 1:**
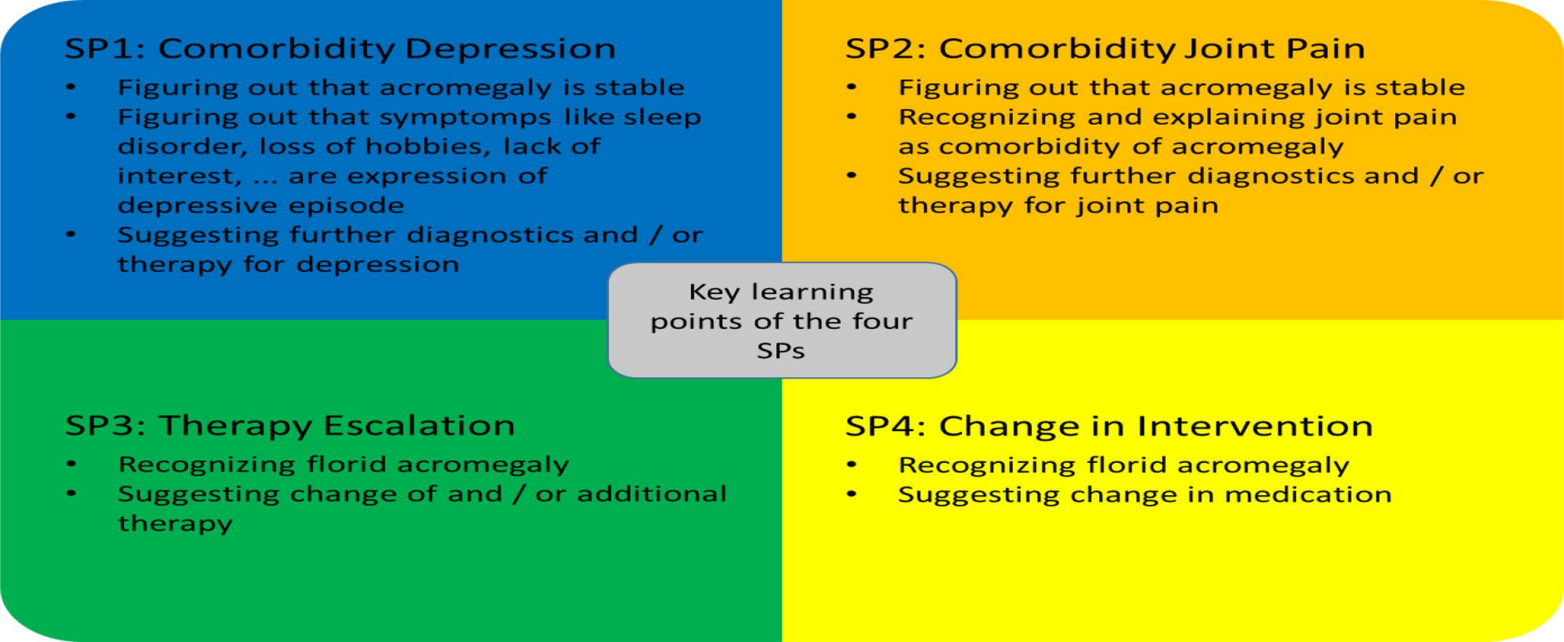
Key learning points of the four SPs

Four different acromegaly case scenarios were scripted for the study by two of the study authors (NU, IKA), both of whom have extensive and long-standing clinical and research experience with the clinical presentation of acromegaly. They were based on a template, provided by the Simulation Persons Program of the Medical Faculty of the University of Duisburg Essen, which contained detailed specifications, not only for the presentation of the medical disease aspects, but also for the entire patient role. For example, the scripts also contained instructions on how to react to certain conversational turns and which information should be given (1) voluntarily, (2) only on request, or (3) when feeling sufficiently at ease during the conversation. In each case scenario, a specific medical problem had to be identified by the physicians and reacted to. Twice, florid acromegaly was to be recognized and twice comorbidities (once depression and once acromegaly arthropathy) were to be identified as the patients’ key points of concern. Corresponding learning points were defined for each of the case scenarios (compare Fig. 1). The SPs were trained with theatre educators from the Simulation Persons Centre at Essen University Hospital. Each SP then had an initial online trial consultation with an experienced endocrinologist (BH) to determine if the scripts were complete and coherent and whether the role had been practiced properly. Full case scenarios can be found in the supplementary material.

### Study setting

All participating physicians received a study information letter containing an explanation of the study procedure and additional information on the SPs. They were instructed that they were covering for a medical colleague who was on sick-leave and that they were to be seeing two of his patients in the scheduled video consultation as a substitute. Prior to the conversations they were provided their “sick” colleague’s treatment notes, current and former IGF-I levels, a hospital treatment report on the performed pituitary surgery and radiology report(s) on current and former cranial magnetic resonance imaging (MRI) investigations. For the consultation in which ACRODAT^®^ was to be used as an additional information source, physicians also received the respective SP’s ACRODAT^®^ profile with the respective graphical representations as a print-out. The participating physicians were asked to treat the SPs as real patients, to discuss the state of the disease with them and to suggest a medical procedure. The actors reacted to the physician`s questions on the basis of the predefined script. All consultations were conducted as online consultations and audiotaped for further analysis (see below). All SPs presented the same illness scenario during the course of the study and opened the conversation always with the same sentence.

**Fig. 2:**
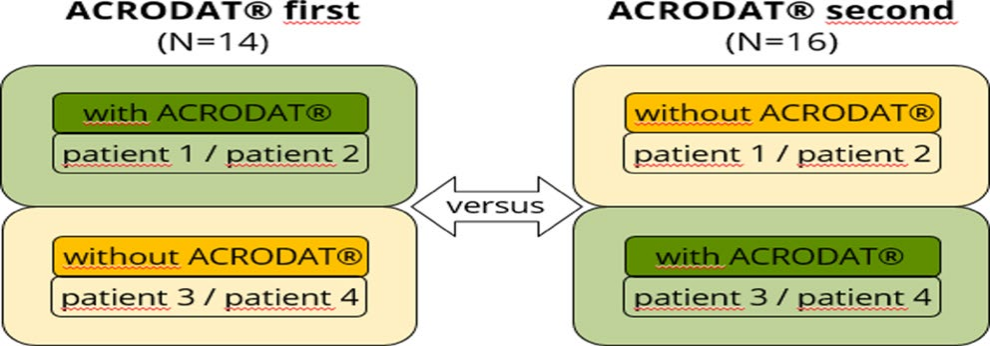
Study design

The project was designed as a cross-sectional control-group design (compare Fig. 2). Physicians were randomly assigned to two groups (ACRODAT^®^ first vs. ACRODAT^®^ second). Each participant interacted with two SPs each (pairing constant), once with and once without ACRODAT^®^ in a cross-over design in two 15–20 min-long online video consultations with the SPs, resulting in 60 conversations, 30 with ACRODAT^®^, 30 without.

After the online consultations physicians also received a self-developed, nine-point Likert-scale questionnaire asking for feedback on the perceived usefulness of the ACRODAT^®^. The SPs answered an eight-point counterpart on their satisfaction with and the quality of the online consultation.

### Analysis

The audio tracks were transcribed using f4x [19]. The resulting transcripts were anonymized and then analyzed by qualitative content analysis with the MAXQDA^®^ (VERBIS GmbH, Berlin, Germany) software [20]. With this method, transcripts are analyzed by sorting all statements from the transcript step-by-step into predefined categories according to a coding manual. To this end, a manual was developed by the research team that defined categories for the analysis of three different aspects of the conversation, specifically: (1) medical content, (2) patient-doctor interaction and (3) shared decision-making. While the categories for medical content were defined by expert opinion, those for patient-doctor interaction were derived using the Calgary-Cambridge Guide [21], a method for structuring clinical interviews. We selected categories suitable to determine a change in communication behavior as a result of the use of ACRODAT^®^. The categories for shared decision-making were defined on the basis of the nine subscales adapted from the Shared-Decision-Making Questionnaire (SDM-Q-9) [22].

For each of the four acromegaly case scenarios, additional categories were included to evaluate, whether case-specific topics were addressed during the consultation. These categories were selected based on the clinical expertise of NU and IKA and can be found in Fig. 1.

Overall, the manual included definitions and coding examples for 27 different categories. Eight of these categories were used to analyze the amount of time spent on the defined topic (e.g. “symptoms”, “QoL”), in the following termed “time-categories”. 19 categories were “yes/no-categories”, used to evaluate dichotomously whether a topic or behavior appeared at all during the conversation (e.g. “asked for relevant comorbidities yes/no”).

The coded MAXQDA files were converted to SPSS 29 for quantitative analysis. For time-categories, the results section reports the percentage (in relation to the respective consultation) of conversation time spent on a topic averaged over all 60 consultations as means and standard deviations (SD). For yes/no categories, the relative frequency of “yes” in all 60 consultations is given in percent. Group comparisons were conducted between the 30 conversations with and without ACRODAT^®^ as well as for gender differences and differences in professional experience with non-parametric measures. Wilcoxon-test and Mann-Whitney-U-test were used for metric variables, whereas McNemar-test was conducted for comparisons of nominal data. No inductive statistics were performed on the 60 patient-doctor interactions, because each subject (physician) held two conversations and is, therefore, included twice in the descriptive analysis. Values reported in parentheses (|≤|) display absolute values, if plus or minus is irrelevant. Values of the Z-distribution are specified. Significance level was set at *p* ≤ .05. Significance values are reported as asymptotic, 2-tailed, if available (Wilcoxon and Mann-Whitney-U-test), otherwise exact (McNemar). We decided against alpha correction in order to decrease the risk of falsely dismissing relevant factors (beta error).

## Results

### Sample

We included *n* = 30 physicians in the study (20 female), of which 17 were in specialist training, 13 were medical specialists in endocrinology. Mean age was 35.53 ± 7.35 (range 27–54 years) and the mean duration of work experience in endocrinology was 6.50 years ± 6.98 (range 0.5–26). The approximate number of treated acromegaly patients each year was on average 17.20 ± 16.80 (range 0–60).

### Patient-doctor interactions (*n* = 60)

#### Time-categories

For the patient-doctor interaction, averaged time-categories over all 60 talks yielded that the greatest part of the conversation contained the topic ‘intervention’ (40.3% ± 22.4), followed by ‘symptoms’ (18.0% ± 10.1), ‘QoL’ (13.2% ± 11.5), ‘IGF-I’ (8.9% ± 5.6), ‘prognosis’ (8.5% ± 9.2), ‘medical history’ (7.6% ± 6.7), ‘tumor’ (7.3% ± 6.9) and ‘comorbidities’ (6.6% ± 8.6).

**Fig. 3:**
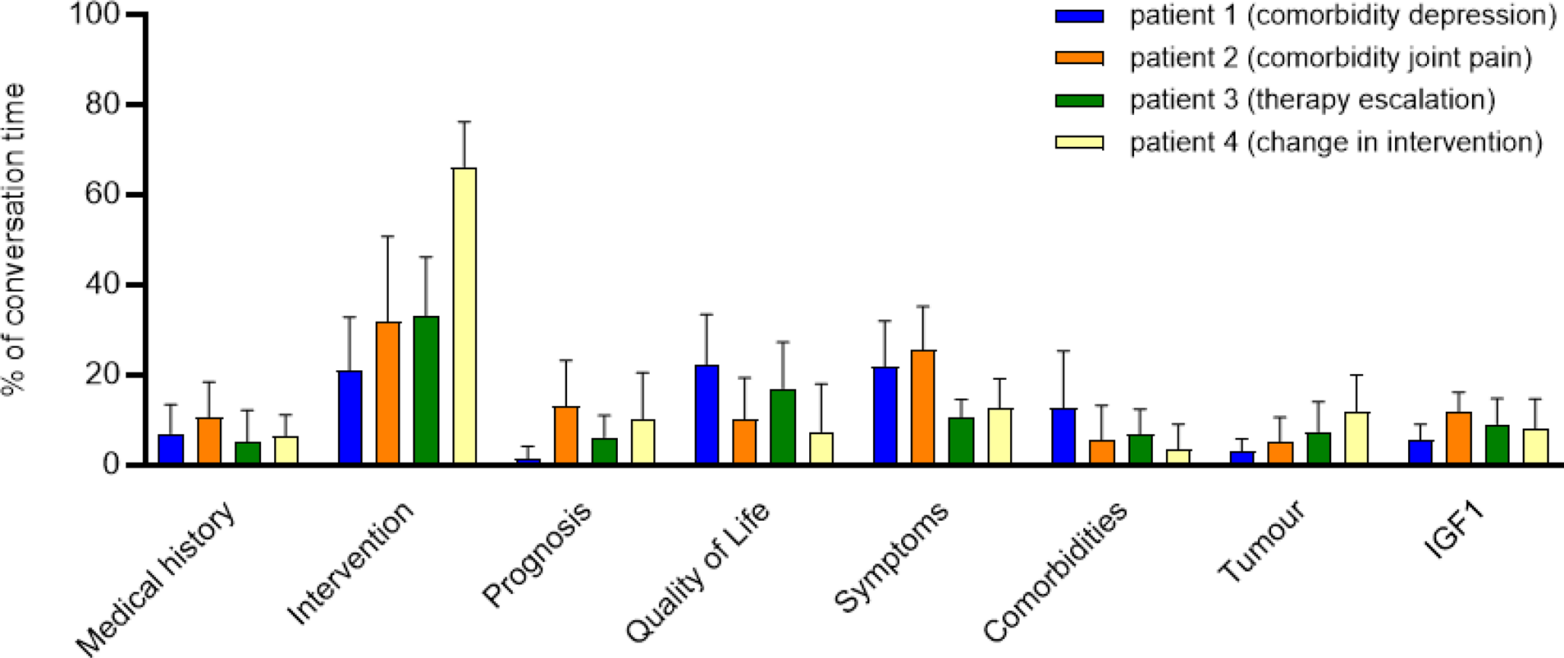
Percentage conversation time divided by the four SPs

The proportion of each category depended strongly on the focus of the patient profile. For detailed results confer to Figure 3.

#### Yes/no-categories

Analysis of the yes/no categories for all four SPs for ***medical details*** showed that the issue of comorbidities was raised in almost half of the conversations, but interestingly those relevant to the particular patient profile were identified in only one third of the consultations.

Concerning ***interaction***, doctors listened attentively to patients in 81.7% of the consultations. In almost every conversation an opinion on the disease activity and an explanation for this opinion was given.

***Shared decision-making*** in the sense of reaching an agreement with the patient for further procedure was achieved in in 98.3% of the conversations. The patient’s desired involvement in the decision, however, was only asked in 18.3% of cases. For detailed results confer to Table 1.

**Table 1:** Percentage of yes in yes/no-categories in all 60 conversations

Yes/no-categories	Percentage yes
**Medical details**	
Asked about hidden problems	31.7%
Recognized hidden problems	18.3%
Asked about relevant comorbidities	45.0%
Recognized relevant comorbidities	33.3%
**Interaction**	
Obtained attentive listening	81.7%
Structured conversation logically	48.3%
Used explicit categorization	18.3%
Gave opinion on disease activity	96.7%
Provided reasons for the opinion on disease activity	91.7%
Made sure that there were no more unanswered questions	70.0%
**Shared decision-making**	
Made clear that a decision needs to be made	18.3%
Asked how the patient would like to participate in the decision	18.3%
Gave information that there are different treatment options	75.0%
Explained the advantages and disadvantages of the treatment options	43.3%
Helped the patient to understand all the information	55.0%
Asked which treatment option they prefer	35.0%
Weighed the different treatment options together	41.7%
Selected a treatment option together	45.0%
Reached an agreement with the patient for further procedure	98.3%

Concerning the specified learning points for the four SPs, stable acromegaly was recognized in the two patients with comorbidities (patient 1 comorbidity depression / patient 2 comorbidity joint pain) with different frequencies (66.7% vs. 100%). In patient 1, recognition of stability (66.7%) and explaining lack of need for therapy change (58.3%) were almost identical in percentage, whereas in patient 2 there was a difference of more than 30.0% in this regard. The respective relevant comorbidity was mentioned explicitly in 66.7% (depression) vs. 94.4% (arthropathy) of the talks. In only 25.0% of conversations it was explained to patient 1 that the current symptoms are an expression of the comorbidity depression. 50.0% of the physicians suggested a therapy for treating the depression, while 83.3% made a suggestion for treating the joint pain.

The fact that acromegaly was florid in patients 3 (therapy escalation) and 4 (change in intervention) was recognized in 83.3% and 100% of the talks. In line with this, a change of therapy was suggested in 91.7% and 100%.

**Fig. 4:**
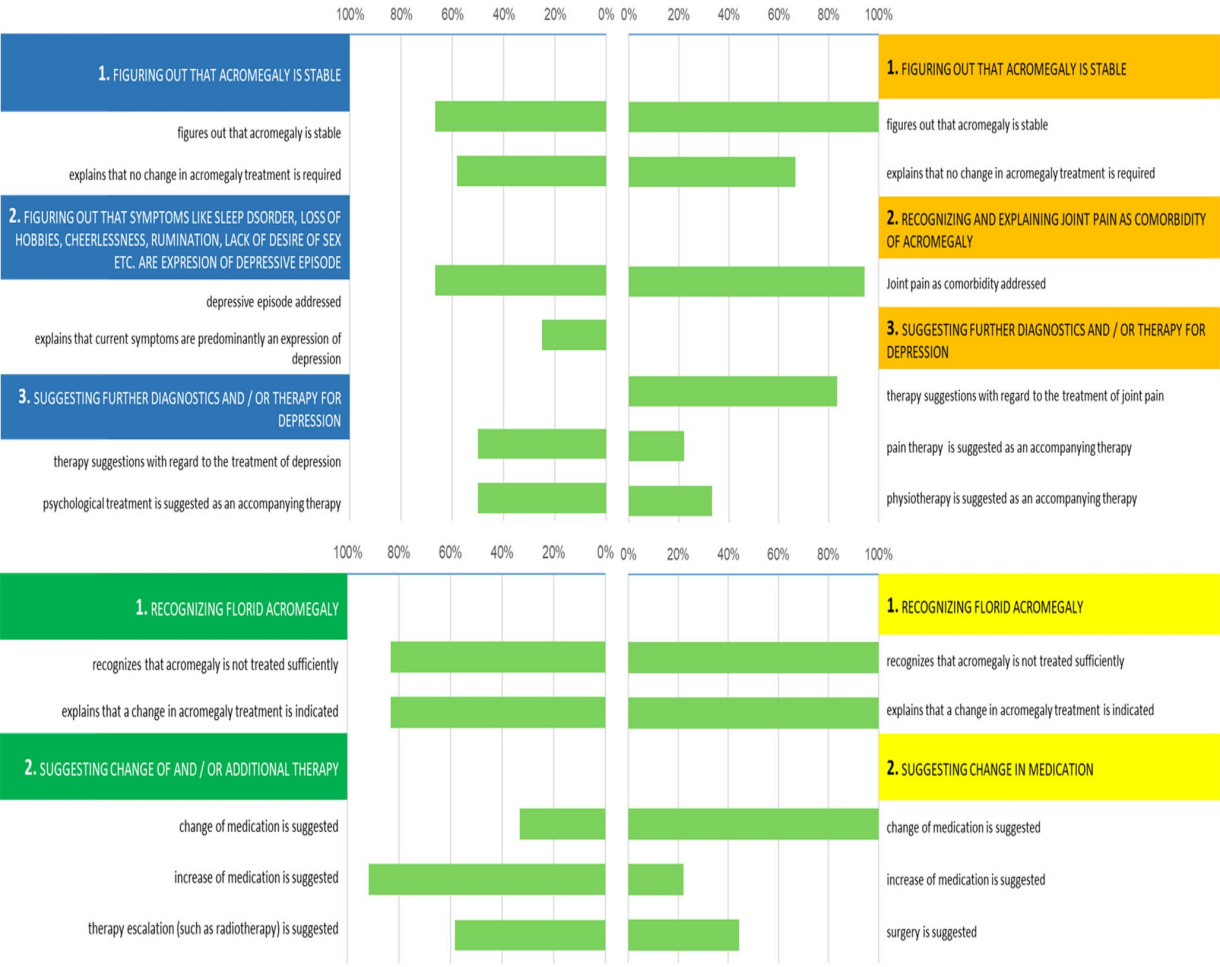
Specific categories of the four SPs with superordinate learning points and their mentioning in the talk

For detailed results confer to Fig. 4.

*SP1: depression*

As depression was the least frequently recognized and addressed comorbidity, all case specific yes/no-categories for patient 1 were analyzed in detail, including differences with respect to the (non-)presence of the ACRODAT^®^ profile (see Fig. 5 in supplemental material).

Symptoms of depression were far more recognized in the conversations without ACRODAT^®^ than with the tool, whereby the criterion for addressing the patients’ discomfort about the appearance, shame about nose and feet, lack of desire for sex, mother´s depression and rumination was met in less than half of the talks overall. Accordingly, the depressive episode was mentioned more often without ACRODAT^®^ (41.7%) than with the tool (25.0%). On the other hand, in a larger proportion of the talks with than without ACRODAT^®^ (16.7% vs. 8.3%) it was explained that the current symptoms are predominantly not of a physical nature, but an expression of the depressive episode. A therapy suggestion regarding the depressive episode was made in 25.0% of the conversations with and without the tool each. Only in 25.0% of the interviews with ACRODAT^®^ and in 41.7% of the conversations without ACRODAT^®^ it was recognized that the acromegaly is stable. While 91.7% (50.0% without vs. 41.7% with ACRODAT^®^) understandingly accepted the patients’ wish for further MRI diagnostic, only 16.7% (all with ACRODAT^®^) declined this request.

### ACRODAT ^®^ tool (n = 30, across subjects)

The Wilcoxon Test yielded no significant differences comparing the time-categories of the conversations (e.g. medical history, QoL) with and without ACRODAT^®^ tool (all Z≤ |1.129|, *p* ≥ .259).

No significant differences in the yes/no-categories were found using the McNemar test, meaning that the ACRODAT^®^ tool did not influence the quality of the conversations in terms of medical details, interaction and shared decision-making (all *p* ≥ .065).

### Gender differences and professional experience of participants

There were no gender differences of the physicians concerning the conversation parts (all Z≤ |1.408|, *p* ≥ .159).

Comparing the differences in the conversation parts between two groups of physicians divided by the median number (= 12.5) of acromegaly patients treated per year, there was a significant difference in the percentage of the conversation about medical history (Z=-2.426 *p* = .015) with physicians who treat more acromegaly patients spending more time on the exploration of the medical history; all other topics did not differ significantly (all Z≤|1.390|, *p* ≥ .165).

Similarly, there were no significant differences in percentage of the conversation topics between physicians that were medical specialists in endocrinology and those still in education (all Z≤ |1.507|, ≥p.132).

### Rating of the ACRODAT® tool by physicians

83.3% found the profile provided by the software tool clear and in logical order and about half of the physicians stated that they found the tool helpful in detecting comorbidities (53.3%) and symptoms that were not addressed (66.7%). Nevertheless, less than half of the physicians would use the tool in their own medical practice (40.0%). For detailed results confer to Fig. 6 in supplemental material.

Order effects of ACRODAT^®^ first or second were checked; the tool was rated worse for all 9 aspects if used in second place, with significant differences for 2 of these aspects (Z≤|2.083|, *p* ≤ .037).

Comparing physicians in medical education and medical specialists in endocrinology concerning the rating of the software tool, all of the rating categories did not differ significantly (all Z≤|1.560|, *p* ≥ .119).

### Rating of the interactions by the SPs

The simulation persons´ subjective ratings indicated that they were very satisfied with the conversations. With regard to the order of ACRODAT^®^ first or second, the SPs showed a better evaluation of the conversation if ACRODAT^®^ was used first (all Z≤|2.245|, *p* ≤ .025). However, rating with vs. without ACRODAT^®^ yielded no significant differences in the categories ‘attentive listening’, ‘empathy’, ‘comprehensibility’, ‘responding to questions’, ‘involving the patient in the decisions’, ‘taking time for the patient’, ‘expertise’ (all Z≤|1.453|, *p* ≥ .146). For detailed results confer to Fig. 7 in supplemental material.

## Discussion

The present study is the first to evaluate shared decision-making in patient-doctor interactions using the ACRODAT^®^ tool in a randomized control-group design with simulated acromegaly patients. Here, we assessed the extent to which shared decision-making was embedded in the conversation, as well as detection of psychological and physical comorbidities. Furthermore, influence of the ACRODAT^®^ tool on meeting these conversation goals was addressed. The need for more sensitive interactions and understanding support by professionals was expressed by acromegaly patients before [3]. This patient-centered approach and shared decision-making are inextricably linked [15].

The results in Figs. 4 and 5 clearly show that the physicians adapted their interviewing behavior to the respective case and that the thematic focus was set according to the relevance for each case. This suggests that the cases were well constructed and realistic and that the setting was appropriate for exploring the questions of the study, namely if the ACRODAT^®^ tool supports physicians in making treatment decisions to achieve better biochemical disease control and in detecting relevant comorbidities, but also to enable a more holistic approach.

All interactions were rated positively by the SPs, indicating overall high conversational skills of the physicians. Table 1 shows the categories in which the doctors performed particularly well and those in which there is room for improvement.

Although agreement was reached on the treatment goal in most cases, many aspects of shared decision-making remained unfulfilled, suggesting that patients could be more involved. Only in 18.3% of the conversations, patients were actively asked how they would like to participate in the decision. Physicians struggle with the implementation of shared decision-making, because of the expected increase of consultation time [23]. In the present study, the time factor was not applicable, because online video consultation were previously scheduled to last 20–30 min. Still, the SP was asked which treatment option he or she preferred only in 35.0% of the conversations. The agreement reached for further procedure in 98.3% might therefore be an agreement rather based on the lack of contradiction of the patient than the active participation in the decision-making process or misinterpretation of preferences of the patient [23, 24]. Since shared decision-making helps to increase patient adherence for different types of diseases [25, 26], it is questionable whether the patients would actually have been adherent (in the long term). The practice and the competencies of shared decision-making therefore seem to be of great importance and should be further extended. However, so far, there is not enough evidence for the effectiveness of interventions for increasing the use of shared decision-making [27], implicating that further research is needed.

Our data show that recognizing comorbidities, especially those of a psychological nature, is a challenge, even for medical specialists in endocrinology. Poorly recognized or treated comorbidities including maladaptive coping strategies could be an explanation for patients’ persistently poor QoL, as shown in several studies [28, 29]. Figure 4 shows that physicians spent significantly less time discussing possible interventions with patients with superficial comorbidities than with the patient for whom the focus was on changing the treatment of acromegaly.

Interestingly, depression is mentioned more often without the tool, but is more clearly diagnosed with the tool. The detection of depression as a comorbidity in medical diseases is aggravated due to symptom overlap [30]. Perhaps, the visualization of the symptoms in the ACRODAT^®^ tool shed a light on the time course of symptoms and disease activity in the depressive patient.

Physicians often do not know what interventions to suggest for psychological comorbidities, which is problematic because it is crucial for a comprehensive treatment offer. In the current study, 50.0% of the physicians suggested a therapy for treating the depression, 83.3% made a suggestion for treating the joint pain.

A central point of our research was the evaluation of the ACRODAT^®^ tool. No significant differences were found between the consultations with and without ACRODAT^®^. This contradicts the original assumption that the tool would influence clinical decision-making by enabling a change of focus of the interaction and allowing more capacity for shared decision-making – maybe because the tool is primarily aimed at recognizing the disease activity of acromegaly, whereas the greater challenge lies in the differentiation and management of comorbidities. According to this assumption, Marazuela et al. found that ACRODAT^®^ detected disease activity in 51.8% of their 111 examined patients [6], reflecting a benefit slightly above chance. Further, although 83.0% of physicians of our study found the tool useful and logical, and 50.0% said it was helpful in recognizing comorbidities, the evaluation did not show improved recognition of these comorbidities. The use of guidelines seems to counteract meeting the needs and preferences of the patients [24]. Since ACRODAT^®^ can be seen as a rule-guiding tool, it could also result in these mentioned negative effects. In fact, only 40.0% of the participating doctors would use ACRODAT^®^ in their practice.

Limitations of the present work include the small sample size, which is probably due to the limited capacity of physicians in their daily routine. In addition, it was not possible to recruit physicians who already used ACRODAT^®^. We did not balance the gender combination of the interviewees, so there was an overrepresentation of female physicians. The strength of our work is that we used a randomized control group design with blinded analysis of the use of the ACRODAT^®^ tool. The trained actors were able to convey the concerns of acromegaly patients in a very authentic and structured way, without unexpected deviations from the protocol, which allowed for good comparability.

Our study represents a new paradigm for direct observation of patient-doctor interaction for research purposes, applied for the first time in endocrinology. This paradigm offers great research potential and can be applied to other diseases, disciplines and research questions. Also, the results of our study could be of great use in the training of residents. It is important that doctors recognize and treat overlapping comorbidities, such as depression and headache. Ideally, treatment networks or focus centers could be set up to ensure optimal treatment by implementing improved detection and management of comorbidities and patient-centered communication.

The results of our study do not indicate a noticeable benefit of ACRODAT^®^. However, the program offers potential due to the combination of *patient-reported outcome and / or experience* and *clinician-reported outcome measures* – both recommended by guidelines for the treatment of acromegaly – and should therefore be further developed with regard to the differentiation of comorbidities. ACRODAT^®^, like the other tools mentioned in the introduction, was developed before the era of artificial intelligence (AI). With the new technical possibilities, not only a better differentiation might be possible in the future, but also concrete intervention proposals.

## Electronic supplementary material

Below is the link to the electronic supplementary material.

Supplementary Material 1

Supplementary Material 2

Supplementary Material 3

Supplementary Material 4

Supplementary Material 5

## Data Availability

Any data not published within the article will be shared in an anonymized manner at request from any qualified investigator.
