# Supplementary material for: Shared decision-making and detection of comorbidities in an online acromegaly consultation with and without the Acromegaly Disease Activity Tool ACRODAT® using the simulated person approach
Source: Pituitary. 2024 Sep 25;27(5):545–54. doi: 10.1007/s11102-024-01460-6 (PMC11513722; doi:10.1007/s11102-024-01460-6)
Supplement: Supplementary file 1 — Supplementary Material 1 [file 11102_2024_1460_MOESM1_ESM.docx]

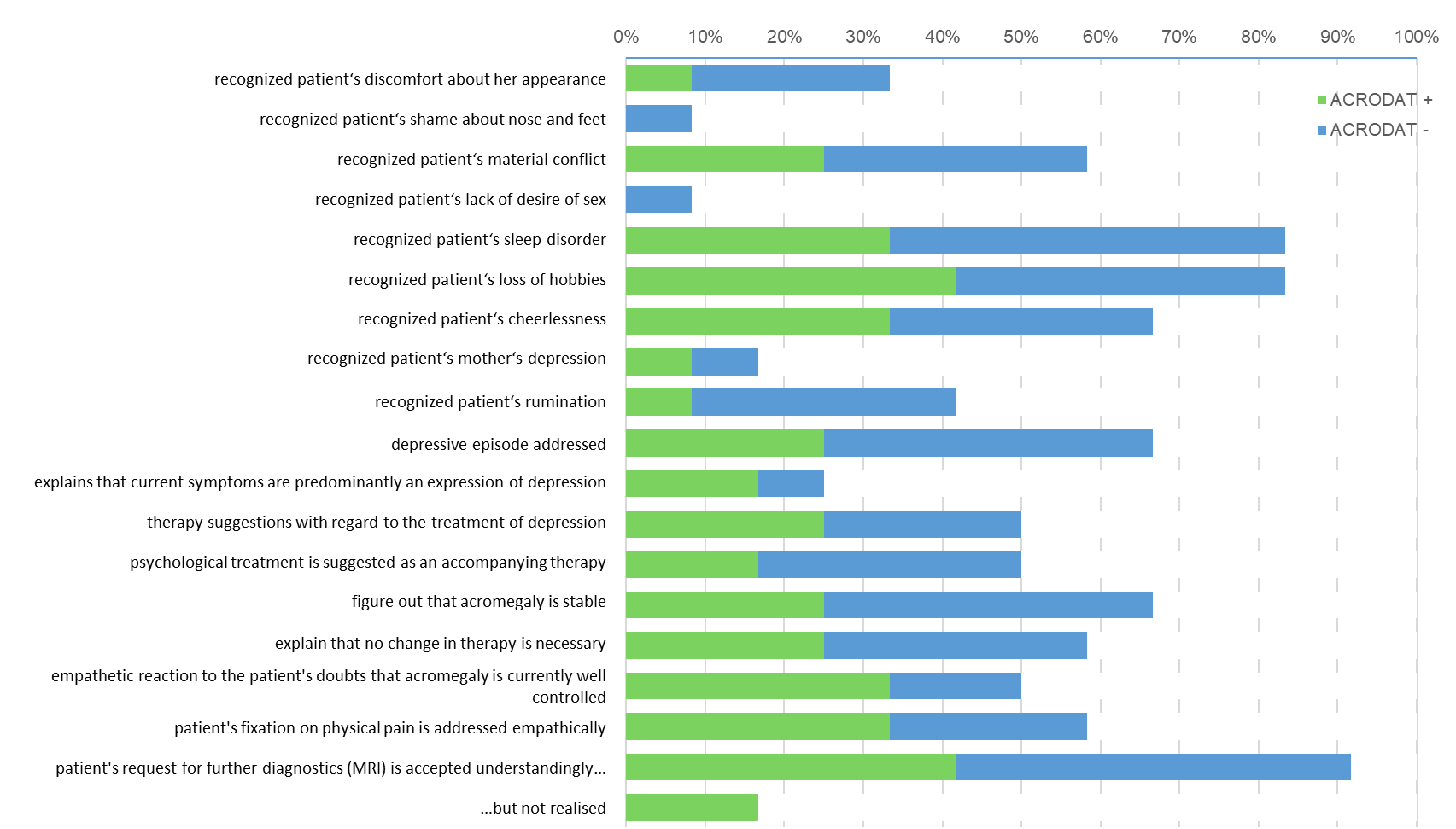


Fig. 5 Specific categories of patient 1 and their mentioning in the talk with and without ACRODAT® tool.


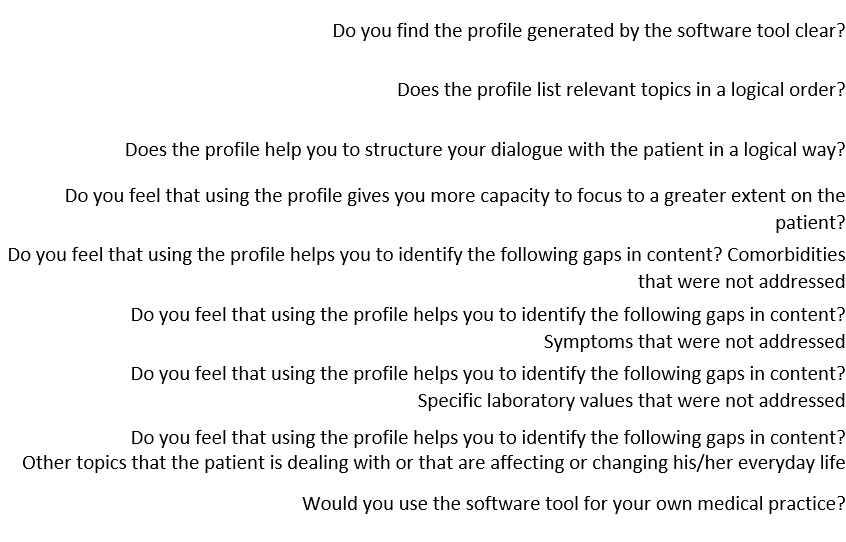


Fig. 6 Ratings of the ACRODAT® software tool by the 30 physicians

Fig. 7 Ratings of the interactions by the SPs; the two bars for each question represent the presence (filled bar) or absence of ACRODAT® (striped bar).
