# Supplementary material for: Shared decision-making and detection of comorbidities in an online acromegaly consultation with and without the Acromegaly Disease Activity Tool ACRODAT® using the simulated person approach
Source: Pituitary. 2024 Sep 25;27(5):545–54. doi: 10.1007/s11102-024-01460-6 (PMC11513722; doi:10.1007/s11102-024-01460-6)
Supplement: Supplementary file 2 — Supplementary Material 2 [file 11102_2024_1460_MOESM2_ESM.docx]

**Profile of ACRODAT patient Brigitte Mueller (SP 1)**

Call duration: 20-30 minutes

**General characteristics**

Name: Mueller, Brigitte

sex: female

Age: 59 years, born 06/24/1961

Appearance: Well-groomed patient. The facial features are coarsened, the nose bulbous, the lower jaw prominent. The hands are widened, the individual fingers appear thick and clumsy. The voice is surprisingly deep.

Other relevant features:

Mrs. Mueller is a trained seamstress, married and has 2 grown-up children. She has not practiced her profession since 2014, but was still tailoring for herself as a hobby until the fine motor work became difficult for her due to changes in her hands and joint pain and took the joy out of the hobby.

After being diagnosed with acromegaly in 2019, Mrs. Mueller was initially shocked, was afraid of dying and had even considered taking "one last trip" before undergoing surgery. However, thanks to the endocrinologist, she quickly got an appointment for surgery with a board-certified pituitary surgeon and had the operation at the Klinik am Park in Siebenhausen in southern Germany. Unfortunately, the tumor was so large at the time of the operation, which was performed through the nose, that it could not be completely removed. Since the operation, Mrs. Mueller has required drug treatment in the form of injections, which are injected into the gluteal muscle once a month by her GP. The treatment is successful. The regularly measured level of the hormone IGF-I (insulin-like growth factor I, which indicates whether the residual tumor is still active) is constantly within the normal range. Some of the symptoms of the disease, such as the swelling in her fingers or the loud snoring, have regressed well. As a result, she was able to sew lighter objects (e.g. tablecloths, cushions, skirts) again from time to time after the treatment for acromegaly and enjoyed sewing again. Recently, however, she no longer feels like doing this activity. She has been feeling worse and worse emotionally for the past six months and is experiencing "unprecedented" dull and pressing headaches that wrap around her head like a ring (before the operation, she only rarely had headaches, and then more in the form of a pain directly behind her forehead). Her knee joints and neck also hurt. She is chronically tired and not very productive, she can only manage her household with difficulty. Sometimes some things, such as dusting, are left undone. She is increasingly dissatisfied with her appearance, suffers from difficulty falling asleep, lack of joy and listlessness. When she can't sleep, she broods a lot. She withdraws, no longer wants to go out or meet up with friends because she feels she is being stared at because of her appearance. Her husband has been wanting more sexual activity in the marriage for some time and also more contact with relatives and friends. The different wishes lead to frequent arguments and are currently developing into a full-blown marital crisis.

Place of consultation:

Video consultation with the treating endocrinologist (= hormone specialist) (m/f/d)

Motivation of the consultation:

Attending a routine appointment planned well in advance. The hope is to get an explanation and a treatment offer for the many complaints.

Opening the interview

"Here I am, Mr./Mrs. Doctor, and I have to tell you: my acromegaly has gotten much worse."

Current ailments:

Headaches, cheerlessness and lack of drive, dissatisfaction with your own appearance, brooding, no desire for sex, chronic fatigue, sleep disorders, increasing joint pain.

Medical history:

5 years before being diagnosed with acromegaly in 2019, Mrs. Mueller noticed increasing pain in her fingers, which grew from year to year and mainly occurred at night. In the morning, she could no longer hold the toothbrush in her hand in the bathroom. Tears welled up in her eyes from the pain. She also noticed increasing swelling of her face and hands and heavy sweating. Both her GP and gynecologist attributed this to the onset of the menopause; even before the acute symptoms began, the patient's periods were infrequent and irregular. The skin had changed and was leathery; the GP had had difficulty inserting the needle through the thick skin, which had been totally embarrassing for her. In retrospect, Mrs. Mueller had also noticed enlarged hands and feet, her shoe size had increased from size 39 to size 42 and her wedding ring no longer fitted. Mrs. Mueller attributed this to increasing osteoarthritis. An MRI scan of the cervical spine was arranged to investigate chronic neck pain. By chance, a tumor of the hypophysis (= pituitary gland in the brain) was found. Further clarification revealed evidence of a growth hormone-producing pituitary adenoma, which was growing invasively in the left cavernous sinus (= dilated venous space belonging to the cerebral blood vessels). This was followed by transnasal transsphenoidal (=through the nose) partial surgery of the tumor in a renowned neurosurgery department. As the tumor could not be completely removed by the operation, a drug therapy with a somatostatin analogue (=growth hormone replacing) was initiated. This improved the symptoms of florid acromegaly such as sweating and snoring, and the IGF1 level (IGF1 = insulin-like growth factor 1) was also within the normal range at the last check-ups. The last MRI of the pituitary gland was performed six months ago and showed the known small tumor remnant in the left cavernous sinus without any change in findings. Due to her illness, she visits an endocrinologist (hormone specialist) every 6 months. The injections required every month are given by the GP because the journey to the endocrinologist, who practices in the next larger town, is too far.

Social history:

Trained seamstress, no longer working since 2014, husband is a self-employed lawyer, regular family income, one adult daughter aged 28, one son aged 34, no grandchildren. The daughter lives in Greifswald, the son lives with his Portuguese partner in Cuimbra. It is a burden for Mrs. Mueller that she has little personal contact with the children. Because of the coronavirus pandemic, she hasn't visited either of them for a long time.

Family history:

Mother died of a stroke at the age of 82, several depressive phases (e.g. postpartum depression, renewed depressive phase at around 50 at the beginning of the menopause), which were mainly characterized by listlessness and were (partly) well treated with medication. Father died at the age of 78 after a heart attack. One brother, 54 years old, healthy.

Medication history:

Until the diagnosis of acromegaly Candesartan 16 mg ½ - 0 - 0 - 0 tablet (against high blood pressure).

After pituitary adenoma surgery, additional octreotide LAR 30 mg intramuscularly once a month (=growth hormone inhibitor). The injection is given by the GP because the journey to the endocrinologist is too far.

Difficulties during the interview/examination:

In the examination situation, the patient is fixated on physical pain, especially joint pain and headaches, complains, does not believe that the acromegaly is currently well treated, but fears renewed tumor growth and urges further diagnostics (a new cranial MRI?). She reports listlessness and fatigue. She often feels unwell because of her appearance.

Behavior during the conversation:

The response latencies (response speeds) are prolonged, the facial expressions are less pronounced, she rarely makes eye contact and fidgets with her hands.

Data that is only mentioned when explicitly asked for:

Marital conflict as an exacerbated stress situation, sleep disorders, loss of hobbies (no longer tailoring), cheerlessness, family history (mother's depression), rumination.

Things that are only told if there is an appropriate atmosphere during the conversation:

No desire for sex, makes further comments about her appearance and the associated shame, e.g. she is uncomfortable with the enlargement of her nose, describes it as a boozer's nose although she doesn't drink alcohol at all. She rarely dares to go to the shoe store because her feet are so wide that she can only find "weird orthopaedic shoes for grannies".

Materials for the practitioner:

Discharge report from the neurosurgery clinic

MRI findings postoperative

Current pituitary laboratory

Other (learning points):

In the doctor-patient consultation, the practitioner should ideally work out that the acromegaly is being treated stably and does not require a change in therapy. However, the symptoms of a depressive episode should be recognized and addressed by the practitioner and further treatment should be offered. Ideally, the patient can be explained that the current symptoms are not predominantly of a physical nature, but an expression of the depression, and she agrees to be referred to a psychiatrist, neurologist or psychotherapist (m/f/d). The main point is that the psychological symptoms is addressed in some way, that an appropriate treatment is offered and that the symptoms are not attributed exclusively to the acromegaly.
