# Supplementary material for: Shared decision-making and detection of comorbidities in an online acromegaly consultation with and without the Acromegaly Disease Activity Tool ACRODAT® using the simulated person approach
Source: Pituitary. 2024 Sep 25;27(5):545–54. doi: 10.1007/s11102-024-01460-6 (PMC11513722; doi:10.1007/s11102-024-01460-6)
Supplement: Supplementary file 3 — Supplementary Material 3 [file 11102_2024_1460_MOESM3_ESM.docx]

**Profile ACRODAT patient Gerd Wagner (SP 2)**

Call duration: 20-30 minutes

**General characteristics**

Name: Wagner, Gerd

Gender: male

Age: 52 years, born 04/15/1969

Appearance: Well-groomed and deliberate, somewhat resigned-looking, soft facial features, yet coarsened-looking, wears horn-rimmed glasses to distract the gaze of others from what he considers his too-large nose, dressed in a light-colored shirt and pleated trousers

**Other relevant characteristics** (e.g. *social status/body posture/body language/emotional situation of the patient - fears and worries, as well as attitude towards the illness*):

Mr. Wagner is a primary school teacher. He has worked for years at the same school in Ruselbach in a well-off neighborhood where there are few social conflicts, but the children sometimes come across as very precocious and the parents like to confuse their offspring's know-it-all attitude with giftedness. Thanks to his even-tempered nature and quiet, natural authority, however, he usually has his classes under control and enjoys his work. The diagnosis of acromegaly was both a shock and a relief for him. He had been suffering from symptoms caused by acromegaly for years before the diagnosis and is still afraid that - despite two operations on the pituitary adenoma and medication that is now working well - the changes that have already occurred, particularly to his joints, will become even more painful and debilitating in the future. He has not had good experiences with orthopaedic specialists, as they did not recognize his diagnosis and were unable to offer him any helpful treatment. On the other hand, he knows that there is no point in withdrawing and hiding and is trying to give his life as much normality as possible.

Place of consultation:

Video consultation with the treating endocrinologist (= hormone specialist) (m/f/d)

Motivation of the consultation:

Attending a scheduled routine appointment. Mr. Wagner wants to address his concerns about his joint problems and wonders whether his acromegaly is actually being treated optimally or whether other medication could help him better.

Opening the interview

Good morning doctor. Have you already looked at my current blood values?

Current ailments:

Pain in both shoulders and hands. He has difficulty writing on the blackboard with his arm raised for long periods of time. Despite wearing comfortable shoes, his feet often hurt so much in the evening that he needs a foot bath. Standing for long periods also causes difficulties because his back hurts.

Medical history:

Mr. Wagner was never really ill until he was forty, apart from colds and minor aches and pains, such as a sprained right ankle. But shortly after his fortieth birthday, he started to feel worse. At first, he couldn't understand why. While he had never suffered from back pain before, it began to hurt more and more frequently. But it was his shoulders that were causing him the most pain. They started to really hurt, initially only when working overhead, such as writing on the blackboard, but later also spontaneously and especially at night. He initially consulted an orthopaedist, who diagnosed impingement syndrome (pinching of a muscle tendon and the bursa between the acromion bones) and prescribed acupuncture. He attributed his back pain to standing a lot in his job as a teacher and his lack of sporting activity. When the pain in his shoulder did not improve, the orthopaedist diagnosed calcium deposits in the shoulder and suggested mobilization of the shoulder joint under anaesthetic. However, on the advice of a friend - and because his knees were also starting to hurt - Mr. Wagner consulted a rheumatologist. She listened to the symptoms and asked about other symptoms that Mr. Wagner had not yet noticed - such as a change in facial features or increased snoring. In retrospect, Mr. Wagner was able to confirm both, including a change in shoe size, which had increased from a size 44 to a size 46 in recent years. The rheumatologist then arranged for a blood test and referred Mr. Wagner for an MRI. In fact, a large hypophyseal adenoma (benign tumor of the pituitary gland) was found and Mr. Wagner was referred directly to a neurosurgeon. During the first operation at a nearby hospital (St. Lukas Neurosurgery in Ruselbach), the chief surgeon operated himself, but was only able to remove a small part of the tumor. The endocrinologist treating Mr Wagner, whom he saw for the first time after the operation, was dissatisfied with the result and the still high growth hormone and growth factor (IGF-I) levels and arranged an appointment for Mr Wagner at a specialist pituitary clinic (Klinik am Park, 71770 Siebenhausen). Here, the tumor was removed in 2018 except for a small, technically inoperable remnant. He was already feeling much better immediately after the second operation. His face swelled up and his snoring was gone. Unfortunately, it was not possible to eliminate the entire over-secretion of growth hormones through the operation, but his endocrinologist was able to put him on a growth hormone inhibitor (Lanreotid Autogel), which he tolerates well. He regularly comes to the endocrinologist's office for injections. Even though he was initially relieved that his condition finally had a name, he is still worried about his joint problems, which are sometimes more and sometimes less pronounced. He has not yet learned to live with them and is afraid that the wear and tear caused by acromegaly will progress and he will become increasingly immobile in everyday life.

Social history:

Married, no children. Wife works as the manager of a car dealership. As both spouses have a lot of contact with other people at work, they value their togetherness in the evenings and at weekends, even though they have friends and family with whom they like to meet up from time to time. Mr. Wagner enjoys reading and cooking. He is not so keen on sport, but he knows that he should exercise more.

Family history:

Mother 83 years old, severely impaired vision due to glaucoma, therefore living in a retirement home, father died two years ago at the age of 82 as a result of lung cancer. A younger brother, living in the same town, 48 years old and fit.

Medication history:

None until the diagnosis of acromegaly.

After pituitary adenoma surgery Lanreotide (trade name Somatuline Autogel® , growth hormone inhibitor), one 90 mg injection deep subcutaneously every 4 weeks. Additionally a medication against high blood pressure (Candersartan 8 mg, 1 tablet in the morning).

Difficulties during the interview/examination:

None, actually. However, due to his status as a private patient, there is an underlying fear of receiving examinations or therapies that are not effective, but are only offered to him because the subsidy pays for them. In his opinion, he had this experience with his orthopaedist.

Behavior during the conversation:

Friendly, quiet, reserved, but not shy.

Data that is only mentioned when explicitly asked for:

Specification of the type and impairment caused by the joint complaints:

Shoulder pain emphasized on the right, dull, pressing, no radiation into the arms, worsening with movement, especially overhead activities, improvement with warmth. Physiotherapy has not yet been prescribed because Mr. Wagner does not currently have an orthopaedist. He does not currently do any physical activity or exercises on his own. Back pain combined with morning stiffness, difficulty getting going, feeling "as if I'm breaking through in my back" after exertion or prolonged standing.

Things that are only told if there is an appropriate atmosphere during the conversation:

Mr. Wagner is very concerned about increasing restrictions in mobility, which he attributes to the fact that acromegaly was not recognized for so long and could therefore lead to irreversible damage to the joints.

Materials for the practitioner:

Discharge report from the second neurosurgical clinic

MRI findings preoperatively, after the first operation and currently (after revision surgery)

Endocrinologist's notes on previous consultations and previous drug treatment with IGF-I values.

Other (learning points):

In the doctor-patient consultation, Mr. Wagner should be told that the acromegaly is well treated and does not require a change in therapy. However, the practitioner should address the comorbidity of the joint pain and make therapy suggestions (e.g. renewed orthopaedic treatment, initiation of pain therapy, prescription of physiotherapy, etc.).
