# Supplementary material for: Shared decision-making and detection of comorbidities in an online acromegaly consultation with and without the Acromegaly Disease Activity Tool ACRODAT® using the simulated person approach
Source: Pituitary. 2024 Sep 25;27(5):545–54. doi: 10.1007/s11102-024-01460-6 (PMC11513722; doi:10.1007/s11102-024-01460-6)
Supplement: Supplementary file 4 — Supplementary Material 4 [file 11102_2024_1460_MOESM4_ESM.docx]

**Profile of ACRODAT patient Melanie Jansen (SP 3)**

Call duration: 20-30 minutes

**General characteristics**

Name: Jansen, Melanie

sex: female

Age: 56 years, born 06/23/1965

Appearance: Short gray hair, sturdy, fashionably dressed, slightly protruding chin, lipstick, fashionable necklace and earrings, not quite slim. Chubby fingers with eye-catching rings.

**Other relevant characteristics** (e.g. *social status/body posture/body language/emotional situation of the patient - fears and worries, as well as attitude towards the illness*):

Ms. Jansen was a freelance journalist. She was known in the industry as a dynamic and creative woman who successfully produced short films for a local television station on all kinds of topics and also regularly published columns in the magazine "SIE!", a business and career magazine for women. The acromegaly disorder has led to a significant career break. She is now unable to work and draws money from her occupational disability insurance. She is now trying to stabilize herself again and live with her illness, but is unsettled by the many health problems of the last 11 years and the fact that her illness is still not well controlled.

Place of consultation:

Video consultation with the treating endocrinologist (= hormone specialist) (m/f/d)

Motivation of the consultation:

Attend a scheduled routine appointment. The current blood values and further therapies are to be discussed. At her last appointment, it was already discussed with her that radiotherapy might be necessary if her blood values continue to fall outside the normal range.

Opening the interview

Good morning, Mr./Mrs. Doctor. Is the therapy finally working?

Current ailments:

Dissatisfaction with the appearance, dents at the injection sites of the Pegvisomant injection (medication for the treatment of acromegaly), still not feeling like living in a "normally functioning body".

Medical history:

Until the age of 45, Ms. Jansen was known as a dynamic and creative woman who successfully produced short films for a local TV station on all kinds of topics and also regularly published columns in the magazine "SIE!", a business and career magazine for women. Then her body changed. She noticed water retention in her hands and face, her periods stopped and she became more apathetic and put on weight, despite all her attempts to lose weight. Her voice became deeper and she often woke up in a cold sweat at night. She no longer wanted to be seen in front of the camera and withdrew more and more from professional life. A number of doctors she consulted put her symptoms down to the menopause. Her family doctor advised her to undergo psychotherapy, while her gynecologist recommended herbal preparations. She spoke to the psychotherapist about her abnormal tiredness and her loss of drive and creativity, only to be told that creativity simply diminishes from the age of mid-forties and that she had to come to terms with it. Ms. Jansen's younger sister, who lives in Canada and came back to Germany for a visit after several years, was horrified by Ms. Jansen's appearance. "Your face and fingers are so swollen, you look like you regularly get into fights!" was her greeting when Mrs. Jansen picked her up from the airport. She urged the nurse to visit the doctor again. Mrs. Jansen's family doctor was already annoyed, partly because he now had more than 20 medical reports in his file from colleagues, none of whom could make any sense of his patient's reported symptoms. After discovering that Ms. Jansen's sugar levels were elevated, he referred her to a diabetologist. However, the attending doctor was initially not interested in the weight gain or the sugar level, but asked about an increase in shoe size and whether the rings still fitted. This was followed by a series of blood tests and an MRI of the head, which confirmed the diagnosis of acromegaly, caused by a benign tumor of the pituitary gland. This was followed by neurosurgical removal of the tumour in 2018 at a clinic specializing in pituitary surgery (Klinik am Park, Siebenhausen). Because the pituitary tumor had already grown into the bone at the base of the skull (clivus), it could not be completely removed. Mrs. Jansen's illness could therefore not be cured by the operation. She was told relatively soon after the operation that she would need lifelong treatment with medication. Her symptoms improved only gradually after the operation. She has now been treated at an endocrinology center for three years. She initially had a growth hormone inhibitor, which she was injected with once a month. The dose was gradually increased. Then she needed an additional medication, which she has to inject under the skin every day. Her growth hormone and growth factor (IGF-1) levels are still not within the normal range. She is also not yet able to work again. At least her disability insurance is now paying out, so she no longer has to worry about her livelihood.

Social history:

Living alone, no children. Ms. Jansen's partner of many years had separated from her in the course of the illness because he could no longer cope with the physical and psychological changes in his former girlfriend. She became increasingly socially isolated as the disease progressed. However, after being diagnosed with acromegaly, she became more cheerful again, also thanks to the support of other patients she met in a local self-help group. She is once again researching and writing smaller articles on hormone diseases for this group and the national self-help network.

Family history:

Father died 12 years ago at the age of 72 after a car accident, mother 82 years old, sprightly, lives in southern Spain. A younger sister, 50 years old, living in Canada and married.

Medication history:

Until the diagnosis of acromegaly, only food supplements and vitamin preparations.

After surgery on the pituitary adenoma, octreotide (trade name Sandostatin® , growth hormone inhibitor), one injection every 4 weeks intramuscularly in increasing doses, most recently 40 mg/ every 4 weeks. In addition, she has been taking pegvisomant (trade name Somavert® , another growth hormone inhibitor) for 2 years, which has to be injected under the skin daily. She takes Metformin for her diabetes and Candesartan 8 mg in the morning for her high blood pressure.

Difficulties during the interview/examination:

Lack of trust in doctors due to the long illness odyssey.

Behavior during the conversation:

Vacillating between resignation and fighting spirit. Then the journalist in her comes through, who also asks investigative questions.

Data that is only mentioned when explicitly asked for:

Details of disease activity such as swelling in the hands, nocturnal snoring, sweating.

Things that are only told if there is an appropriate atmosphere during the conversation:

Fear that the acromegaly will not respond to the next treatment, but also fear of radiation, which she associates with the treatment of malignant diseases.

Materials for the practitioner:

Discharge report from the neurosurgery clinic

Current MRI findings

Current IGF-I value and preliminary values from regular endocrinology consultations.

Notes from the endocrinologist on previous consultations and existing drug treatment.

Other (learning points):

In the doctor-patient consultation, Ms. Jansen should be informed that the acromegaly is not yet sufficiently treated and that further therapy escalation (e.g. change of medication or radiotherapy) is necessary.
