# Supplementary material for: Shared decision-making and detection of comorbidities in an online acromegaly consultation with and without the Acromegaly Disease Activity Tool ACRODAT® using the simulated person approach
Source: Pituitary. 2024 Sep 25;27(5):545–54. doi: 10.1007/s11102-024-01460-6 (PMC11513722; doi:10.1007/s11102-024-01460-6)
Supplement: Supplementary file 5 — Supplementary Material 5 [file 11102_2024_1460_MOESM5_ESM.docx]

**Profile of ACRODAT patient Richard Bergmann (SP 4)**

Call duration: 20-30 minutes

**General characteristics**

Name: Bergmann, Richard

Gender: male

Age: 47 years, born 02/11/1974

Appearance: Well-trained, athletic, angular facial features, protruding chin, gaps between teeth, slightly protruding ears, dressed in a T-shirt and tracksuit

**Other relevant characteristics** (e.g. *social status/body posture/body language/emotional situation of the patient - fears and worries, as well as attitude towards the illness*):

Mr. Bergmann is a fitness trainer and has run his own gym for many years. He is physically fit, takes care of his diet and still has a bit of a chip on his shoulder that he didn't take the symptoms of acromegaly seriously for so long and only - in his opinion too late - did his own research into the causes. Due to the long undiagnosed illness, he has learned not to trust doctors unconditionally, but to critically question medical findings and suggestions. He wants to be perceived by them as a partner at eye level and has a self-confident, albeit respectful demeanor. He has learned to come to terms with his diagnosis of acromegaly by looking up disease-related information and regaining an active lifestyle. He enjoys traveling and is looking forward to going on another long backpacking vacation in Asia once the corona pandemic is over.

Place of consultation:

Video consultation of the treating endocrinologist (= hormone specialist) (m/f/d)

Motivation of the consultation:

Attending a routine appointment planned long in advance. Mr. Bergmann is glad that the appointment is finally taking place, as he is experiencing increasing physical complaints again.

Opening the interview

"Hello Doc! I'm worried. There's another lodger up there. I think he's causing trouble."

Current ailments:

Increased sweating again, hands become stiffer due to water retention, onset of headaches - just like before (stabbing in the right forehead area) - but not as bad as before the operation. He also thinks he is snoring more at night again - at least that's what the app he uses to track his sleep says.

Medical history:

Mr. Bergmann had always been very active in sports, but for some years he had noticed that his physical fitness was declining somewhat. He had been noticing for some time that his facial features were slowly changing, as he regularly updated photos of himself and his colleagues for the visitors' board at the gym. His dentist pointed out to him that the position of his teeth was slowly changing, although the dentist did not present this as a problem. Looking back, his fingers and hands were also getting wider, but he put this down to regular training with weights and equipment. Although he had never been a headache person, a year ago he began to experience increasingly severe, stabbing headaches and facial pain, particularly in the area of his right forehead and right eye, which responded poorly to pain medication. A good friend of his, with whom he had shared a hotel room on a weekend trip, approached him about unbearable snoring and breathing interruptions. Mr. Bergmann realized that something was seriously wrong and googled his symptoms on the Internet. He then asked his GP if he could have a hormone test as he thought he had a pituitary tumor. After the blood test showed initial evidence of an excess of growth hormone and the insulin-like growth factor IGF-I, Mr. Bergmann was referred to the endocrinology department in Silberbrunn, where the diagnosis of acromegaly was confirmed with further blood tests and an MRI of the head was arranged. He was operated on at a renowned clinic for pituitary surgery in southern Germany (Klinik am Park, 71770 Siebenhausen). Although no clear residual tumor was seen in postoperative MRI scans of the head, the postoperative IGF-I levels were repeatedly slightly elevated, so that his treating endocrinologist (m/f/d) initiated drug therapy with cabergoline (a drug for the treatment of acromegaly that binds to dopamine receptors and thus reduces the release of growth hormone from the pituitary gland) under the suspected diagnosis of a residual tumor not visible in the MRI. This drug is only approved for the treatment of acromegaly if the postoperative IGF-I levels are not more than 2-fold above the age norm. It originally had a good effect on Mr. Bergmann and was also well tolerated. For about six months, however, he has again noticed symptoms that he also had when he had active acromegaly, albeit not as pronounced. Shortly before his appointment with the endocrinologist (m/f/d), he had an MRI scan of his head. He translated the MRI findings with the help of Google and is concerned because "there is a newly recognizable widening of the right cavernous sinus compared to the last preliminary examination, which is suspicious of a residual/recurrent tumor". The IGF-I level measured by the endocrinologist is currently 3.5 times higher than the age norm.

Social history:

Single, trained car mechanic, but gave up his job and has been running his own gym for 10 years. No steady partner, but good social environment.

Family history:

Mother 77 years old, treated for high blood pressure, otherwise healthy. Father 82 years old, heart problems, has already had two heart stents and has to take several heart medications, although he was also very sporty throughout his life. Two older sisters, 51 and 49 years old, no significant previous illnesses.

Medication history:

None until the diagnosis of acromegaly.

After pituitary adenoma surgery, cabergoline (trade name Dostinex® , mild growth hormone inhibitor) 0.5 mg twice a week at night.

Difficulties during the interview/examination:

The patient wants the findings to be explained in detail and to be involved in the decision-making process. They do not want to be patronized and make no secret of the fact that, although they see the endocrinologist (m/f/d) as an expert, they want to make the decision on further treatment together with her/him.

Behavior during the conversation:

Friendly, self-confident, but also concerned about new tumor growth and the associated consequences.

Data that is only mentioned when explicitly asked for:

Great concern about increasing restrictions on lifestyle and health due to re-existing active acromegaly, especially fear of becoming as heart-diseased as his father.

Things that are only told if there is an appropriate atmosphere during the conversation:

Concern that another drug treatment for acromegaly may lead to lifestyle restrictions (e.g. in terms of injections that need to be given regularly (every 4 weeks) and increased medical check-ups)

Materials for the practitioner:

Discharge report from the neurosurgery clinic

MRI and MRI findings preoperative, postoperative and current

Current pituitary laboratory

Notes from the endocrinologist on previous consultations and existing drug treatment.

Other (learning points):

In the doctor-patient consultation, the practitioner should work out that the acromegaly is florid again and requires a change in therapy. Specifically, it should be suggested that the patient stop taking Cabergolin and that another growth hormone inhibitor medication be used instead.
